# Predictors of ageing-related decline across multiple cognitive functions

**DOI:** 10.1016/j.intell.2016.08.007

**Published:** 2016

**Authors:** Stuart J. Ritchie, Elliot M. Tucker-Drob, Simon R. Cox, Janie Corley, Dominika Dykiert, Paul Redmond, Alison Pattie, Adele M. Taylor, Ruth Sibbett, John M. Starr, Ian J. Deary

**Affiliations:** a Department of Psychology, The University of Edinburgh, Edinburgh EH8 9JZ, United Kingdom; b Centre for Cognitive Ageing and Cognitive Epidemiology, The University of Edinburgh, Edinburgh EH8 9JZ, United Kingdom; c Department of Psychology, University of Texas, Austin, TX 78712-1043, USA; d Alzheimer Scotland Dementia Research Centre, The University of Edinburgh, Edinburgh EH8 9JZ, United Kingdom

**Keywords:** Cognitive ageing, Cognitive decline, Longitudinal, Structural equation modeling

## Abstract

It is critical to discover why some people's cognitive abilities age better than others'. We applied multivariate growth curve models to data from a narrow-age cohort measured on a multi-domain IQ measure at age 11 years and a comprehensive battery of thirteen measures of visuospatial, memory, crystallized, and processing speed abilities at ages 70, 73, and 76 years (*n* = 1091 at age 70). We found that 48% of the variance in change in performance on the thirteen cognitive measures was shared across all measures, an additional 26% was specific to the four ability domains, and 26% was test-specific. We tested the association of a wide variety of sociodemographic, fitness, health, and genetic variables with each of these cognitive change factors. Models that simultaneously included all covariates accounted for appreciable proportions of variance in the cognitive change factors (e.g. approximately one third of the variance in general cognitive change). However, beyond physical fitness and possession of the *APOE* e4 allele, very few predictors were incrementally associated with cognitive change at statistically significant levels. The results highlight a small number of factors that predict differences in cognitive ageing, and underscore that correlates of cognitive level are not necessarily predictors of decline. Even larger samples will likely be required to identify additional variables with more modest associations with normal-range heterogeneity in aging-related cognitive declines.

## Highlights

• We modeled aging-related cognitive decline from age 70 to 76 in a narrow-age sample.
• A general factor explained 48% of variance in cognitive change.
• Of numerous covariates, few significant predictors of change emerged.
• Cognitive ageing is likely a process with few large-effect predictors.

## 1 Introduction

Populations are ageing in industrialized societies across the world: it is forecast that, by 2020, there will be as many people over 60 years old as people under 15 years old in the global population (Harper, 2014). Among many challenges, this demographic shift brings with it a greater proportion of the population in cognitive decline, one of the most feared—and economically costly—sequela of ageing (Wimo, Jönsson, Bond, Prince, & Winblad, 2013). Cognitive decline is pervasive even in healthy older adults without dementia (Boyle et al., 2013), and is associated with a reduction in the ability to perform everyday functions, make important decisions, and thus live independently (Boyle et al., 2012, Tucker-Drob, 2011a). Consequently, research efforts to understand, and thus potentially mitigate, the effects of normal cognitive decline are of great practical importance. A fundamental question in the field of cognitive ageing, addressed in this study, asks: Why do some people's cognitive abilities decline more steeply than others'?

Many risk and protective factors for cognitive ageing have been proposed, both by epidemiological researchers and in the popular media. They span genetic, health, physical, lifestyle, and socio-demographic contributors (e.g. Brunner, 2005, Salthouse, 2014). A systematic review of the literature (Plassman, Williams, Burke, Holsinger, & Benjamin, 2010) concluded that factors such as smoking status, diabetes mellitus, and the *APOE* e4 genotype were associated with more cognitive decline, whereas better physical health and some kinds of cognitive training were protective against it. However, the often-contradictory studies meant that the overall evidence was rarely assessed to be strong in either direction. Importantly, variables that are known to affect the intercept or starting point of cognitive decline (such as education, which has been suggested to boost cognitive ability levels into later life; Banks and Mazzonna, 2012, Clouston et al., 2012, Stern, 2002) do not necessarily affect its rate of progression (Tucker-Drob, Johnson, & Jones, 2009).

This lack of consistent findings may stem from the fact that each individual study only examined a small number of potential correlates. Due to mutual intercorrelations, correlations involving some putative risk factors may be partially or fully artefacts of associations driven by other, often unmeasured, factors. If only small numbers of predictors are included in a study, these confounding relations will be missed. Studies that simultaneously include a large number of potential predictors can more effectively reduce this type of confounding. For the present analysis, we took a multivariate approach, examining a detailed longitudinal dataset. We included a cross-cutting, yet carefully selected, set of potential socio-economic, somatic fitness, health, and genetic predictors of cognitive ageing, all of which had previously been investigated in the literature.

Many—though by no means all—previous studies have also been limited by small cognitive batteries, often with single indicators of cognitive domains. Sometimes, tests that are intended to screen for dementia, such as the Mini-Mental State Examination (Folstein, Folstein, & McHugh, 1975) are used to indicate cognitive function. Such tests are less sensitive to subclinical levels of, and changes in, cognitive functions. The reliance on such instruments can attenuate, obscure, or even reverse the sign of associations with cognitive change (Wang, Zhang, McArdle, & Salthouse, 2009). Moreover, the reliance on changes in specific cognitive tests, rather than more general factors, limits generalizability: it is unclear whether the test-specific results obtained will hold for other tests or domains. This may also be a source of inconsistency across studies, if risk factors associate with decline in some cognitive tests or skills, but not others.

The current study estimated latent factors of cognitive levels and cognitive changes from a broad assortment of highly sensitive cognitive tests developed to capture both normal range and clinical-range variation in four distinct domains: visuospatial ability, memory, crystallized ability, and processing speed (Tucker-Drob, Briley, Starr, & Deary, 2014). These latent factors represent shared variation among different cognitive abilities, and shared variation in their changes across time. By examining changes in latent factors, rather than in individual tests, we ensured that our results pertained to the theoretical cognitive constructs in which we were interested, rather than to idiosyncratic aspects of specific tests. We also examined correlates of a *general* factor of cognitive change, which previous research has found to account for between approximately one half and two-thirds of the variance in later-life cognitive decline across many tests (e.g. Ghisletta et al., 2012, Lindenberger and Ghisletta, 2009, Reynolds et al., 2002, Tucker-Drob, 2011b, Tucker-Drob et al., 2014).

We report findings from the Lothian Birth Cohort 1936 (LBC1936), a sample of older individuals (initial *n* = 1091) recruited at mean age 70 years who had taken an intelligence test at mean age 11 in 1947. They were tested on a varied battery of cognitive tests at age 70, and these were repeated at ages 73 and 76. The eighth decade of life is an important time for studying cognitive ageing, because of the doubling in the risk of dementia that occurs across it (Matthews & Brayne, 2005).

**Fig. 1:**
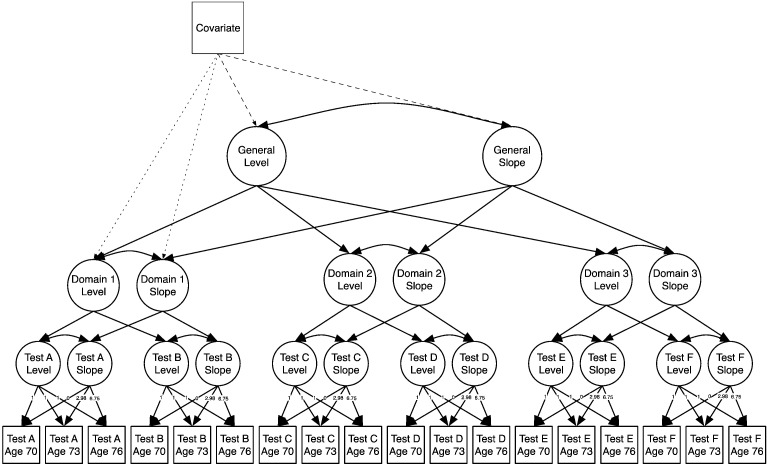
Simplified diagram of the ‘factors of curves’ model. A growth curve, including a latent level and slope factor, was estimated for each individual cognitive test, and these intercepts and slopes were factor analyzed in a hierarchical model which included domain-level factors and higher-level general factors of both level and change. We then tested for potential covariate relations with the general factors (dashed lines) and, in a separate model, the domain factors (dotted lines). This diagram only shows 3 domains with 2 tests per domain; the full model included 4 domains with at least 3 tests per domain. The basis coefficients (loadings on the slopes) were set to 0, 2.98, and 6.75 to precisely represent the amount of time passing between assessments. Although not represented in the path diagram, the means of the test-specific latent levels and slopes were all freely estimated.

We modelled many potentially predictive factors first individually, and then in concert. These included socio-demographic (sex, education, parental occupational status, own occupational status, deprivation of the residential area), physical fitness (lung function, walking speed, and grip strength), genetic (*APOE* e4 status), lifestyle (smoking and body mass index), and health (cardiovascular disease, hypertension, and diabetes) variables, as well as childhood cognitive ability. By including this latter variable in the model, we were able to test the extent to which any associations of the covariates with later-life cognitive ability were confounded by prior ability. To assess change within old age, we used a ‘factors of curves’ model (McArdle, 1988), which involved modeling the slope of longitudinal change in each test in a latent growth curve model, then loading these slopes onto cognitive domains (visuospatial ability, crystallized ability, memory, and speed), which are themselves loaded onto the general factor of cognitive change (Fig. 1). The predictors were tested for their associations with this general factor, and with the domain factors.

Note that a previous study (Tucker-Drob, Briley, et al., 2014) examined a similar set of questions in data from the LBC1936, but was limited by the inclusion of only two waves of testing (subsequent data were not available at that time). It could not, therefore, use growth curve modeling, relying instead on latent change score modeling. The present study thus doubles the time-frame of the previous paper (from approximately three to six years). It includes two extra potential predictors (Body Mass Index and alcohol consumption). It assesses the associations of the potential predictors with general factors of level and change, in addition to the domain-level factors that were investigated in the previous paper. It also includes a set of four sensitivity analyses—ruling out potential confounders such as dementia status and mood state—that were not included in the previous paper.

Thus, with our combination of a ‘factors of curves’ model and a wide range of covariates, our study was able simultaneously to address the following two questions. First, to what extent do different domains of cognitive ability age together, or seperately? Second, which, if any, variables predict between-person variation in cognitive changes, both in the specific cognitive ability domains and more generally across all cognitive abilities, from age 70 to 76, even after multivariate statistical control for a host of covariates?

## 2 Method

### 2.1 Participants

Three waves of data in older age were available for members of the Lothian Birth Cohort 1936 (LBC1936; Deary et al., 2007, Deary et al., 2012), a narrow-age sample of community-dwelling individuals, living mostly in the Edinburgh and Lothians area of Scotland, UK. All of the participants were born in 1936 and were aged approximately 11 years at the time of the Scottish Mental Survey of 1947, in which most completed the same group test of general intelligence (Scottish Council for Research in Education, 1949). They were followed up in 2004–2007 (wave 1: mean age 69.53 years, SD = 0.83; *n* = 1091, 543 female), again in 2007–2011 (wave 2: mean age 72.49 years, SD = 0.71 *n* = 866, 418 female), and again in 2011–2014 (wave 3: mean age 76.25 years, SD = 0.68; *n* = 697, 337 female). For the present study, 552 participants contributed data on all cognitive tests and all covariates at all three waves, though the models took into account all the available data (see Statistical analysis, below).

The mean time between waves 1 and 2 was 2.98 years (SD = 0.28), and between waves 2 and 3 was 3.77 years (SD = 0.28). From waves 1 to 3, the mean time lag was 6.75 years (SD = 0.31), with a minimum of 5.12 years and a maximum of 8.98 years. The youngest participant at wave 1 was aged 67.61 years, and the oldest participant at wave 3 was aged 77.70 years.

Before participation, all cohort members completed a written consent form. The study was approved by the Multi-Centre Research Ethics Committee for Scotland (MREC/01/0/56; 07/MRE00/58) and the Lothian Research Ethics Committee (LREC/2003/2/29).

### 2.2 Measures

#### 2.2.1 Cognitive tests

##### 2.2.1.1 Later-life cognitive ability

The participants completed a battery of thirteen individually-administered cognitive tests in the same location, and using the same equipment and instructions, at all three waves (Deary et al., 2007). Following previous analyses on this battery of cognitive tests (Tucker-Drob, Briley, et al., 2014), we hypothesized that the tests' scores were grouped into the following four domains.

*Visuospatial ability* consisted of two subtests from the Wechsler Adult Intelligence Scale, 3rd UK Edition (WAIS-III^UK^; Wechsler, 1998a): Matrix Reasoning and Block Design. It also included the Spatial Span (Forward and Backward) subtest from the Wechsler Memory Scale, 3rd UK Edition (WMS-III^UK^; Wechsler, 1998b). The Spatial Span score used here was an average of forward and backward performance.

*Processing speed* was measured using two pencil-and-paper tests from the WAIS-III^UK^ (Symbol Search and Digit-Symbol Substitution), and two tests using dedicated instruments: Four-Choice Reaction time, where participants have to press one of four buttons indicated by a number from 1 to 4 flashed up on an LCD screen (Deary, Der, & Ford, 2001), and Inspection Time, where participants must discriminate between two figures flashed on a computer screen for a variety of durations from 200 ms to 6 ms, then immediately backward-masked. There were 150 Inspection Time trials (10 at each of 15 durations), and the measure we used was the total number of correct responses (Deary et al., 2004).

*Verbal memory* was measured using two subtests from the WMS-III^UK^ (Verbal Paired Associates and Logical Memory), and the Digit Span Backward subtest of the WAIS-III^UK^.

*Crystallized ability* was measured by two tests that involved the participant reading aloud a list of irregular words of decreasing linguistic frequency (and thus increasing difficulty): the National Adult Reading Test (NART; Nelson & Willison, 1991), and the Wechsler Test of Adult Reading (WTAR; Wechsler, 2001). We also included a test of phonemic verbal fluency (Lezak, 2004).

##### 2.2.1.2 Age 11 cognitive ability

Most participants had completed the Moray House Test No. 12, at mean age 11 (Scottish Council for Research in Education, 1949). It is a group-administered, general intelligence/IQ-type test, with a 45-minute time limit. It has a preponderance of verbal reasoning items, and also some numerical and other items. In concurrent validity testing, it correlated about *r* = 0.8 in childhood with the individually-administered Stanford-Binet test (Deary, Whalley, Lemmon, Crawford, & Starr, 2000).

#### 2.2.2 Covariates

A variety of socio-demographic, physical fitness, genetic, lifestyle, health, and mood covariates were measured at wave 1 (in the case of mood, also at waves 2 and 3). All had previously been linked to cognitive ageing, though never all together in the same model.

##### 2.2.2.1 Socio-demographic

Number of years of formal, full-time education was reported at interview at the first testing wave. In addition, three measures of socioeconomic status (SES) were recorded. First, we noted the occupational class of the participant's father's job, rated on a 5-point scale from class I (professional) to class V (unskilled; Office of Population Censuses and Surveys, 1951). Second, we noted the occupational class of the participant's own most prestigious job before retirement (on a similar scale with an extra distinction between manual and non-manual work in class III; Office of Population Censuses and Surveys, 1980). Third, for each person's small area of residence in later life, we obtained a score using the Scottish Index of Multiple Deprivation (Scottish Executive, 2006), a neighborhood-level measure of SES that ranked 6505 geographic areas in Scotland from most to least deprived, based on income, employment, health, crime, and other socio-demographic indicators. This measure was split into eight groupings for the purposes of the current analysis, with higher numbers indicating less deprivation.

##### 2.2.2.2 Physical fitness

Three measures of physical function were taken at the first testing wave. First, lung function was indexed by forced expiratory volume (FEV) in one second (best of three attempts), measured using a Micro Medical Spirometer. Second, time to walk 6 m was recorded. Third, grip strength was measured using a North Coast Hydraulic Hand Dynamometer for the left and right hands, and averaged into an overall index.

##### 2.2.2.3 Genetic

The participants provided samples of blood for DNA extraction. Each participant's apolipoprotein E (*APOE*) e4 status (either one or two e4 alleles present vs. no e4 alleles) was genotyped using TaqMan technology at the Wellcome Trust Clinical Research Facility Genetics Core at the Western General Hospital, Edinburgh.

##### 2.2.2.4 Lifestyle

Smoking status (current smoker/ever smoked vs. never smoked), was also recorded at interview. Alcohol consumption (in grams per week) was recorded from a food frequency questionnaire taken at age 70, as described by Corley et al. (2011).

##### 2.2.2.5 Health

Body Mass Index was calculated in the standard fashion, by dividing the participant's weight (in kg) by their height (in m) squared. Self-reported diagnosis history of three illnesses potentially related to cognitive ageing—cardiovascular disease, hypertension, and diabetes—was recorded at interview as a dichotomous variable (presence vs. absence of the illness).

##### 2.2.2.6 Mood

Mood was measured using the Hospital Anxiety and Depression Scale (HADS; Zigmond & Snaith, 1983), a questionnaire that was completed by the participants at all three waves. The scale includes 7 anxiety-related items (e.g. “I feel tense or ‘wound up’”), and 7 depression-related items (e.g. “I look forward with enjoyment to things”; negatively scored). The participants rated their agreement with each item, or how often they had the particular experience mentioned in the item, on a 4-point scale. These items were summed into a total HADS score at each of the waves that was used in our sensitivity analysis as a time-varying covariate.

### 2.3 Statistical analysis

Data were analyzed using a multivariate latent growth curve approach (McArdle, 2009), implemented in Mplus v7.3 (Muthén & Muthén, 1998–2014) using full-information maximum likelihood (FIML) estimation to take all data into account. The model estimates the overall *level* of each cognitive test (in effect, the intercept at mean age 70 years) and the *slope* of its change across the three measurement waves (in effect, the trajectory between age 70 and 76 years). We used the average time lag between the waves (2.98 years from waves 1 to 2, and 6.75 years from waves 1 to 3) as the path weights for calculation of the slope factor, with the path from the slope factor to the initial wave's test score being set to zero. The resulting latent level and slope factors can then be analyzed as if they were directly measured variables: their organization into higher-order factor structures can be investigated (for this reason, the analysis is known as a ‘factor of curves’ model (McArdle, 1988; Fig. 1)), as can their relations with covariates. See the Supplementary Method section for a description of our calculations of the percentage variance explained at each level of the model.

We first fit a model in which growth curves were estimated for each individual test, while estimating an unstructured covariance matrix in which all levels and all slopes were allowed to correlate with one another. As this model is the least constrained model possible, we used it as a baseline against which to judge the fit of the more parsimonious factors of curves models.

Next, we estimated a single general factor from the growth curve levels for each test. We tested whether it was possible to extract a similar general factor of cognitive change from the growth curve slopes. We tested whether this ‘general factors only’ model had good fit to the data. We then tested whether better model fit could be achieved with a hierarchical structure, in which intermediate domain-specific factors were modelled, first for the levels, and then for the slopes. Model fit was tested using four absolute fit indices: Root Mean Square Error of Approximation (RMSEA; values < 0.06 considered acceptable), Comparative Fit Index (CFI; values > 0.95 considered acceptable), Tucker-Lewis Index (TLI; values > 0.95 considered acceptable), and Standardized Root Mean Square Residual (SRMR; values < 0.08 considered acceptable). Models were compared using the chi-square test and by comparing two relative fit indices: Akaike Information Criterion (AIC) and Bayesian Information Criterion (BIC).

The large number of significance tests in our analyses increases the chance of a false positive result. For that reason, we corrected the *p*-values in our two main models (the models with simultaneous covariate associations with general factors—36 tests—and domain factors—144 tests—respectively) for multiple comparisons using Hochberg's False Discovery Rate (FDR) correction (Benjamini & Hochberg, 1995). Below, we report the results with and without this correction.

## 3 Results

### 3.1 Ageing-related change in cognitive tests

**Fig. 2:**
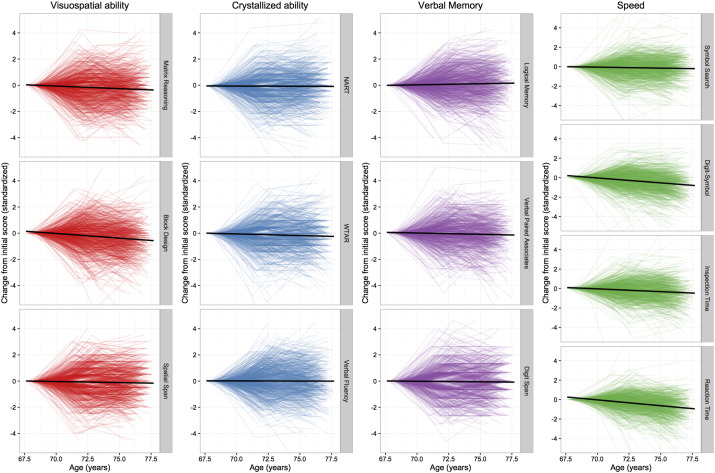
Individual trajectory plots of change scores on each of the cognitive tests with age, grouped by cognitive domain. One colored line is included for each participant, indicating the change from their score at the initial testing wave. The black central line in each plot indicates the mean trajectory. All tests are scored such that lower scores represent poorer performance. Note that, in order to highlight the heterogeneity in change, individual differences in baseline test scores have been subtracted from all individual trajectories.

Descriptive information about the cognitive tests and the covariates, and their zero-order relations with one another, is provided in the Supplemental materials document (Tables S1–S3). We first tested whether there was significant ageing-related mean change in each of the cognitive tests from age 70 to 76. In the baseline model in which all levels and slopes were free to covary, there was a significant, negative mean slope across the three waves in ten of the thirteen cognitive measures. The trajectories for each test (slope means) are detailed, alongside the means and variances for the intercepts, in Table 1, taken as a difference from each individual's baseline score. They are illustrated (purely descriptively, for each individual) in Fig. 2. The three tests that did not show significant mean decline were the National Adult Reading Test, Verbal Fluency, and Logical Memory, the latter of which had a positive (though non-significant) mean slope.

### 3.2 Prediction of study (non-)attendance

As noted above, we used FIML estimation to reduce bias due to missing data. This operated under a ‘missing at random’ assumption (Rubin, 1976), that patterns of missingness in the data were not systematically related to the missing scores themselves, but could be accounted for by other data within the model. To test the extent to which the other variables accounted for study attendance at the two follow-up waves, we ran two logistic regression models with attendance/non-attendance at age 73 and age 76 as the outcome variables, respectively, and all of the predictor variables described above as predictors (we did not include the time lag between waves as a predictor in these models as this information is undefined for participants who did not return).

From these models, we produced the ROC curves shown in Fig. S1 in Supplementary materials. The area under the curve estimates (where 1.00 indicates perfect prediction and 0.50 indicates prediction that is no better than random chance) were 0.644 for wave 2 attendance and wave 2 and 0.640 for attendance at wave 3. Nagelkerke's *R*^2^ showed that the logistic regression model accounted for 6.9% of the variance at wave 2 and 7.5% of the variance at wave 3. Thus, the predictions of attendance were weak, but the predictors did provide a modest amount of additional information which could be used by the FIML algorithm to reduce bias. We thus included all the potential predictors as ‘auxiliary’ variables (e.g. Muthén & Muthén, 1998–2014, p.389) in the models below that estimated the structure of cognitive change. Auxiliary variables are taken into account by FIML even though they are not explicitly included as covariates in the model.

### 3.3 Structure of cognitive change

We compared the fit of alternative models of the structure of cognitive ability levels and changes to one another and to the baseline model of unstructured level-change covariance. In many of our factor models, six of the tests' slopes had specific variances that were near-zero and were thus sometimes estimated as negative, indicating that all variance in change on that test was shared with the domain above them (i.e. they have a standardized loading of 1.0). Therefore, to allow the models to converge on within-bounds estimates, we fixed the specific variance of these six slopes (Spatial Span, WTAR, Verbal Fluency, Verbal Paired Associates, Digit-Symbol Substitution, Choice Reaction Time) to zero in our factor models.

Table 2 reports model fit comparisons of alternative factor models of levels and changes. As is the case for most applications of factor analysis, there was a significant increase in *χ*^2^ misfit associated with all factor models relative to the unstructured (level and slope) covariance matrices (all *p*-values < 0.001). All models, however, fit well by RMSEA, CFI, and TLI indices relative to the covariance matrix of the raw data. In order to gauge local fit of the factor models of levels relative to an unstructured level covariance matrix, and local fit of the factor models of the slopes relative to an unstructured slope covariance matrix, we also calculated RMSEA indices for these comparisons (rightmost column of Table 2). The RMSEA for a general factor model of the levels (Model 2), with no domain-specific group factors, compared to an unstructured level covariance matrix (Model 1) was unacceptable (RMSEA = 0.102), whereas the RMSEA for a hierarchical factor model of the levels (Model 3), compared to the same unstructured level covariance matrix, was good (RMSEA = 0.049). Chi square, AIC, and BIC comparisons also indicated that the hierarchical structure of levels (Model 3) had superior fit to a model with only a general factor of levels (Model 2; *χ*^2^(4) = 1541.51, *p* < 0.001; ΔAIC = 2031.43, ΔBIC = 2011.46).

We went on to make analogous comparisons for the covariance structure of the slopes. RMSEA for a general factor model of the slopes (Model 4), with no domain-specific group factors, relative to a model with an unstructured slope covariance matrix (Model 3), was good (RMSEA = 0.041), but the RMSEA for a hierarchical factor model of the slopes (Model 5) relative to this same unstructured slope covariance matrix was even better (RMSEA = 0.035). *χ*^2^, AIC, and BIC comparisons also indicated that the hierarchical factor model of the slopes (Model 5) fit the data better than a model with only a general factor of slopes (Model 4; *χ*^2^(8) = 58.07, *p* < 0.001; ΔAIC = 42.07; ΔBIC = 2.12), though the difference in BIC, which penalizes more heavily for more complex models, was not as substantial as that for the difference in AIC. Thus, we proceeded with the fully hierarchical model (Model 5) of both levels and slopes for the analyses below.

**Fig. 3:**
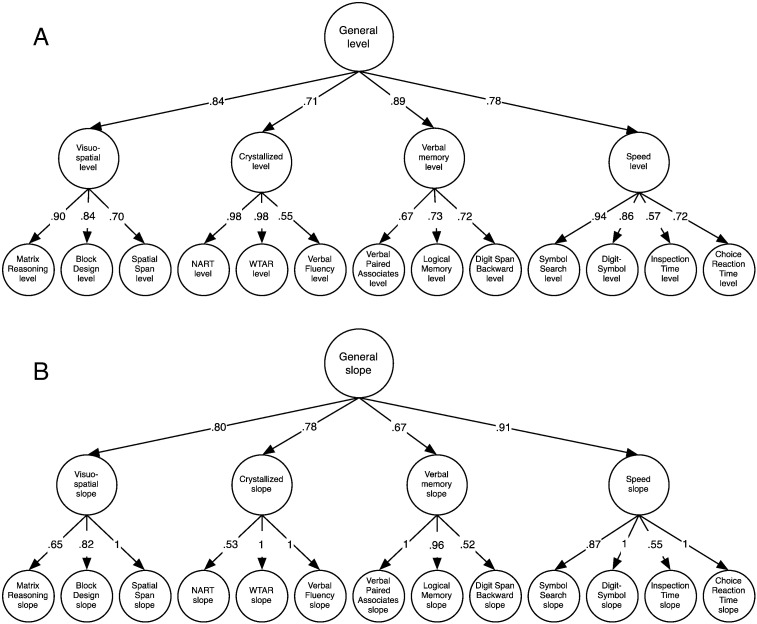
Structural model of cognitive ability levels (A) and slopes (B). The latent levels and slopes of each test are grouped into domains; these domains are themselves grouped under the general factor of cognitive ability. Values are standardized factor loadings. Although the results for level and slope are shown as separate parts of this diagram, they were estimated simultaneously in the model.

For cognitive levels, an average of 40% of the variance in performance on each of the 13 cognitive tests was explained at the general latent trait level, 23% at the domain level, and the remaining 37% at the level of individual tests. Individual tests had high loadings (from 0.57 to 0.98, mean = 0.78) on the latent traits of their respective domains. All four latent cognitive domains had high loadings on the latent general cognitive level factor (from 0.71 to 0.89, mean = 0.81). For cognitive slopes from age 70 to 76, an average of 48% of the variance in declines in performance on 13 cognitive tests was explained at the general level, 26% at the domain-specific level, and 26% at the test-specific level (Fig. 3). The general factors of both level and change accounted for substantial proportions of variance: not only are cognitive ability levels in large part explained by a large general factor, almost half of cognitive change variance between age 70 and 76 was shared: that is, much of the change in the thirteen cognitive tests was due to changes in general cognitive function.

### 3.4 Predictors of cognitive level and change

We next sought to identify predictors of individual differences in cognitive level and cognitive slope, at the level of, first, the general factors and, second, the cognitive domains. To begin with, we regressed just the general factors of cognitive level and slope on all of the covariates individually. This was repeated with all the covariates included simultaneously.

#### 3.4.1 Correlates of later-life cognitive ability level

Next, we addressed the question of what might contribute to differences in cognitive ability level at age 70. The covariates were entered individually alongside the basic factors of age and sex. All of the covariates except sex, time lag between testing, and *APOE* e4 status were significantly associated with the general level of cognitive ability (all results are shown in Table S4 in Supplemental materials). Those LBC1936 participants with better general cognitive function at age 70 were younger when tested, had higher childhood intelligence, were more educated, were from more professional occupational classes, lived in more affluent areas, were fitter (on all three performance indicators), had lower BMI, were less likely to smoke, and were less likely to have cardio-metabolic illness. The four cognitive domains showed a similar pattern of results, with the additional finding that carriers of the *APOE* e4 allele also performed less well on the visuospatial and speed domains. The three illnesses had little association with the verbal memory and crystallized domains levels.

We next ran an intermediate analysis where we entered each covariate alongside age, sex, and age 11 cognitive ability. This model, wherein older age cognitive level was adjusted for childhood cognitive ability (which correlated *r* = 0.786, *p* < 0.001 with later-life general cognitive ability in this intermediate model), effectively tested whether each of the predictors were associated with cognitive change between age 11 and age 70 (at least to the extent to which there was shared variance between the early- and later-life abilities being tested). Many significant predictors of cognitive level in the univariate models were no longer significant (even before correction for multiple comparisons) in this adjusted model, potentially due to their associations with age 11 ability (see Supplementary Table S5). The effect sizes of the covariate associations with the general factor of level were attenuated by an average of 61% (with the largest amount of attenuation—90%—being found for cardiovascular disease), implying a great deal of confounding. The average amounts of attenuation in the domains model ranged from 47% to 64%; Supplementary Table S6 shows the effect size attenuations for each individual covariate. Despite these effect size attenuations, all of the socioeconomic and physical variables retained significant relations with later-life cognitive ability.

After the inclusion of all the covariates, only age, sex, age 11 IQ, education, and forced expiratory volume remained significant correlates of general cognitive ability level (see Table 3 and for full results, Table S7). In the domains model, *APOE* e4 status was significantly negatively associated with Speed, and BMI was positively associated with visuospatial ability (this latter effect, which was in an unexpected direction, was modest, *β* = 0.132). None of the social or health variables remained significantly associated with cognitive ability level after including all the other variables as covariates and correcting for multiple comparisons.

#### 3.4.2 Predictors of later-life cognitive change

Which variables predict differences in cognitive ageing between age 70 and 76? First, we entered covariates individually (alongside age and sex). Those who declined less in general cognitive function from 70 to 76 were less likely to carry the *APOE* e4 allele, and were fitter as assessed using measures of lung function, walking time, and grip strength (see Table S4 in Supplementary materials for all effect sizes). There were small associations suggesting that those with higher childhood cognitive ability and more education had steeper declines. We next examined the ageing slopes of the four cognitive domains. Carriers of the *APOE* e4 allele declined more steeply in Visuospatial ability, Speed, and Verbal Memory. Again, there were associations indicating that physically fitter individuals—especially those with greater grip strength—declined less in cognitive ability, especially in Speed. There was a strong association between better grip strength and maintenance of Crystallized ability with age.

We then tested these associations with cognitive change between age 70 and 76 in the fully-adjusted model, where all covariates were added in concert. Importantly, a large number of the significant associations from the previous model did not survive this mutual adjustment for all other variables. Even fewer survived correction for multiple comparisons. For instance, associations between cognitive change and childhood SES and diabetes were only just below the *p* < 0.05 threshold for statistical significance and thus did not survive adjustment and FDR correction. This was also the case for three associations with change in Visuospatial ability (FEV, walk time, and BMI), four associations with change in Verbal Memory (baseline age, sex, childhood SES, and grip strength) and one association with change in Speed (sex). Results that were significant before FDR correction are indicated in Table S7.

The few surviving associations are shown in Table 3, with the full results shown in Table S7 in Supplementary materials. Carrying the *APOE* e4 allele was associated with more decline in general cognitive ability (effect size *d* = − 0.499, corrected *p* = 0.001), as well as Speed (*d* = − 0.440, *p* = 0.013) and Verbal Memory (*d* = − 0.357, *p* = 0.031). Women tended to decline less in general cognitive ability (*d* = 0.578, *p* = 0.022), and this appeared driven by protective associations with Crystallized ability (*d* = 1.162, *p* = 0.002). There was also an association of grip strength with decline in general cognitive ability (standardized *β* = 0.262, *p* = 0.022)—those with stronger grip declined less, and this was again likely driven by associations with decline in Crystallized ability (standardized *β* = 0.492, *p* = 0.013)—but not with any of the other cognitive factors. The model *R*^2^ values showed that all the predictors together accounted for 80.5% of the variance in cognitive level, and 16.1% of the variance in general cognitive decline.

### 3.5 Sensitivity analyses

We conducted a series of sensitivity analyses to test the robustness of our results for cognitive level and cognitive slope. First, since it has been suggested that effects of *APOE* e4 on cognitive decline may be due only to dementia pathology (Bunce et al., 2014), we removed individuals with possible dementia in two different ways (using a dementia screening instrument [Folstein et al., 1975] and using dementia ascertainment via medical records). Next, we adjusted for low mood at the time of testing—which may result in the appearance of additional cognitive decline—by using the HADS, measuring anxiety and depression as a time-varying covariate. These initial three sensitivity analyses, which are detailed in Supplementary materials, made little difference to the results reported above.

In a fourth and final sensitivity analysis, however, we did find an important difference from our main analysis. Here, instead of including all three of the physical fitness variables (lung function, grip strength, and walk speed) separately in the model, we created a unit-weighted composite of the three variables by summing their *z*-scores. This resulted in an ‘overall fitness’ variable, which was entered as a covariate after removing the individual fitness variables from the model. In the model with general factors, we found that higher scores on this variable were associated with both higher level (standardized *β* = 0.161, *p* = 0.007) and less decline (*β* = 0.368, *p* < 0.001) of cognitive ability; both of the relations survived FDR correction. In the domains model, the ‘overall fitness’ variable was associated with the level of both Visuospatial ability (*β* = 0.225, *p* = 0.004) and Speed (*β* = 0.271, *p* < 0.001), and these survived FDR correction. There were no significant associations with the level of Verbal Memory or Crystallized ability (absolute *β*s < 0.08 *p*s > 0.20). In terms of slope, there were significant associations between overall fitness and the slopes of Speed and Crystallized ability (*β*s = 0.303 and 0.394, respectively; *p*s < 0.003), but the associations with Visuospatial ability and Verbal Memory, while significant in the uncorrected model, did not survive FDR correction (*β*s > 0.225). Overall, more relations with physical fitness were found when using an overall fitness variable. Indeed, using this latent variable, the *R*^2^ value indicated little change in the variance accounted for in cognitive level (81.0%), but a substantial increase in the variance accounted for in cognitive change: 32.0%. Potential explanations for this finding are discussed below.

## 4 Discussion

This study verified the structure of cognitive ageing in the eighth decade of life, and attempted to disentangle the associations with many of its potential predictors. We found that a substantial proportion (48%) of the variation in cognitive decline across thirteen different tests is shared, and that a well-fitting model can be estimated that groups this slope variance into a hierarchy including variance due to individual tests, cognitive domains, and the general factor of cognitive change (the general factor implies that, to a considerable extent, it "all goes together when it goes"; Rabbitt, 1993). After mutually controlling for all the covariates, there were very few associations between the potential predictors and both domain-general and domain-specific ageing-related cognitive declines. This was not the case for cognitive levels: a very large proportion of the variance in the levels could be explained by all the predictors in concert. Thus, our results underscore the difficulty in finding predictors of differences in cognitive ageing, even when using variables that are strong correlates of baseline cognitive ability.

We found a great deal of confounding of possible contributors to levels of cognitive abilities at age 70 years by prior cognitive ability at age 11 years. Early life cognitive ability was not related to cognitive change within the eighth decade (Tables 2 and S8). We tested here for possible ‘differential preservation’ (between-person differences in the gradient of the ageing trajectory) of cognitive functions by a large number of covariates and, though we found this to a small degree, there was much more evidence for ‘preserved differentiation’ (pre-existing between-person differences preserved into later life) of cognitive ability between childhood and older age (Salthouse et al., 1990, Salthouse, 2006). That is, as shown in some previous analyses of this cohort (Deary et al., 2012), brighter children become brighter, healthier, and fitter older adults. This ‘preserved differentiation’ appeared to last into the eighth decade of life.

The most robust and consistent predictor of cognitive change within old age, even after control for all the other variables, was the presence of the *APOE* e4 allele. *APOE* e4 carriers showed over half a standard deviation more general cognitive decline compared to non-carriers, with particularly pronounced decline in their Speed and numerically smaller, but still significant, declines in their verbal memory. The mechanism by which *APOE* e4 may have its effects is currently unclear, but the presence of the allele has been linked to neural characteristics including reduced cerebral blood flow (Thambisetty, Beason-Held, An, Kraut, & Resnick, 2010), hippocampal atrophy (den Heijer et al., 2002), and a thinner frontal cortex (Fennema-Notestine et al., 2011). Our sensitivity analysis indicated that these effects were independent of dementia pathology: that is, the *APOE* e4 allele appeared to relate to normal, not just pathological, cognitive ageing.

Another variable associated with cognitive decline was sex. The previous literature is unclear as to the existence and direction of sex differences in cognitive decline (Barnes et al., 2003, Karlamangla et al., 2009); we found that women had significantly less general cognitive decline than men, mainly centered on Crystallized ability. Unexpectedly, we found that individuals who were older (within the narrow age range) at the initial testing wave tended to age more healthily in terms of their Visuospatial ability across the subsequent six years. There is no obvious explanation for this result, but given the very narrow age range of the sample, we would caution against drawing strong inferences about this effect. One potential explanation is sample selectivity; that is, we may have missed unhealthy older individuals as they were less likely to volunteer for the study, meaning that the oldest individuals who were recruited tended to be healthier. In addition, having a higher age 11 IQ was predictive of more decline in visuospatial abilities in the fully-adjusted model. This may reflect the ‘law of initial value’, whereby those with initially higher abilities decline faster as there is more ‘room’ for them to do so (Wilder, 1957). A similar explanation may apply to the finding that individuals who had undergone more education showed greater decline in their crystallized abilities, though this finding was no longer significant after adjustment for multiple comparisons.

The relation of physical fitness to later-life cognitive change was ambiguous in our analysis. When entered individually, the three fitness predictors (lung function, grip strength, and walk speed) showed few associations with cognitive change. However, when an ‘overall fitness’ variable was estimated across all three fitness indicators, larger associations with cognitive decline were found. This may indicate that broad measures of fitness, reducing the measurement error specific to each individual indicator, are required to detect substantive relations with cognitive function; higher general fitness, and not necessarily scores on specific measures of physical function, may be what is protective against cognitive decline. Physical function measures are among the most potentially modifiable of the covariates we studied. Admittedly, since our results are from observational data, they could not (even if strongly positive) be used to infer that improving physical function, e.g. via exercise, would have concomitant, causal benefits for cognitive ageing (the ostensibly protective effect may be due to individuals having genes that give them both healthy bodily and cognitive systems in old age, for example). Nevertheless, it is of interest to compare these results to the growing body of evidence—some from randomized controlled trials—suggesting protective effects of exercise and physical activity on cognitive change in later life (Lövdén et al., 2013, Sofi et al., 2011; though see Young, Angevaren, Rusted, & Tabet, 2015).

Contrary to some conceptions of ‘cognitive reserve’ (Stern, 2002), we found no evidence for a relation between education (or social class) and the slope of any of the cognitive factors. Our results are therefore in line with some previous analyses that suggest education may contribute to the level, but not to the slope, of ageing-related cognitive decline (Gottesman et al., 2014, Tucker-Drob et al., 2009, Zahodne et al., 2011). We did, however, find an association between earlier cognitive ability and cognitive decline, in that those with higher childhood ability tended to decline more in visuospatial ability. There was no significant effect of earlier cognitive ability on the slopes of all the remaining cognitive measures. Thus, our results were a further test of the previously-asked question regarding whether ‘age is kinder to the initially more able’ (Gow et al., 2012). The answer, from our fully-adjusted model, is ‘no’.

Even with a more liberal significance threshold—that is, without controlling our main analysis for multiple comparisons with the FDR correction—there were still very few significant predictors of ageing-related cognitive change (in the general-factors model, there were 4 significant predictors of change before correction and 3 afterwards; in the domain-factors model, the respective numbers were 14 and 5). Without correction, some variables, including FEV and childhood SES, appeared relevant for some areas of cognitive ageing. After correction, they did not relate to any of the slope factors. Overall, however, it is important to note that our interpretation—that it is difficult to observe predictors of cognitive decline—was not contingent on our strategy of FDR correction: after mutual inclusion in the model, there were very few significant predictors to begin with.

One plausible explanation for our failure to discover a sizable number of predictors of cognitive decline is statistical power. The pattern of our results, where many predictor associations were found with intercept and few with slope, is predicted by simulation studies such as that of Hertzog, Lindenberger, Ghisletta, and von Oertzen (2006), which showed that very high reliabilities and large numbers of measurement occasions, in addition to the well-known sample size issues, are required to achieve adequate statistical power in longitudinal studies. We note that the simulations in that study referred to tests for correlated change, but the same argument applies to the current study. Even though von Oertzen, Hertzog, Lindenberger, and Ghisletta (2010) have shown that including multiple indicators of a latent construct increases a study's power—the current study used a thirteen-test cognitive battery to estimate variation in general cognitive change—it may still have been the case that, had our study been larger in sample size, been carried out across a larger number of measurement waves, or had even greater variance in the change measures, we would have been able to detect other, smaller associations with the predictors.

## Strengths and limitations

The LBC1936 is a narrow-age cohort; this removes the troublesome effect of chronological age that hampers making conclusions about what affects within-person cognitive ageing (Hofer & Sliwinski, 2001). The participants are generally healthy, as well as ethnically, geographically, and culturally homogeneous, and our study thus avoids the possible confounding effects of acute inter-current illness and population stratification. Our study includes a comprehensive range of cognitive tests and covariates, and uses latent cognitive factors to diminish the influence of measurement error.

The above factors improve the robustness of our results, but nevertheless, there are a number of limitations that may affect their generalizability. The sample was somewhat restricted in cognitive ability range and social status, and thus some effect sizes might be under-estimates. The higher social class of our participants means that they were healthier than the general population: it is possible that this explains the lack of any relations of health or lifestyle variables to cognitive change in our sample, though none of these effects were even close to statistically significant in the fully-adjusted model. None of the participants were acutely ill, so the results do not describe cognitive decrements caused by current illnesses. The geographical- and age-specific nature of the sample also means that we should be cautious about generalizing to other groups.

Repeated measurements over time are what enable longitudinal studies to track within-person changes over time, but they also result in accumulating familiarity both with the testing material and with the more general testing situation that may produce practice (retest) effects. Practice effects may have masked some of the cognitive decline that participants experienced. Such practice effects are likely responsible for the slight, non-significant, increase in Logical Memory score across age, as the same story was used at each of the waves and the participants may have remembered aspects of it from the previous wave. Indeed, in a study of short-term retests effect, in which actual cognitive changes are guaranteed to be highly trivial, Salthouse and Tucker-Drob (2008) report the largest retest effects of repeated administrations of episodic memory tests, including Logical Memory. Importantly, although studies that have implemented methods for formally estimating practice effects have found that practice effects are moderate-to-large on average, they indicate that between-person variation in practice effects tends to be minimal (for a review, see Tucker-Drob & Salthouse, 2011). This suggests that correlates of longitudinal changes in cognitive test performance primarily reflect correlates of ageing-related changes in cognitive abilities, rather than correlates of practice-related learning. In addition, the present study will have missed cognitive decline that occurred up to age 70; it is possible that declines over this period are associated more strongly with some of the proposed predictors.

Finally, we used a FIML algorithm in order to use all of the available data under the ‘missing at random’ assumption (Rubin, 1976) that, conditional on the available non-missing data, the pattern of missingness was not systematically related to the unobserved scores on the cognitive tests under study. Our analysis of predictors of dropout indicated that the baseline covariates did not fully account for the patterns of study dropout at subsequent waves (Fig. S1). With FIML, estimates from growth curve models are not expected to be biased as long as the remaining heterogeneity (not accounted for by these covariates) in dropout was either entirely random, or nonrandom but unrelated to the missing cognitive tests scores post-dropout. However, if residual heterogeneity in dropout was either due to the cognitive decline that we were not able to observe (due to the dropout) or due to a set of third variables that are themselves associated with cognitive decline post-dropout the estimates reported here may be biased. Study attrition is, of course, a pervasive problem in ageing research.

## Conclusion

The broad answer to why some people's cognitive skills age better than others is still elusive. Outside of acute illness, dementia, the *APOE* e4 allele, and overall physical fitness, healthy cognitive ageing is likely either to involve factors not studied here, or to be—by analogy with the ‘polygenic’ model of the genetics of complex traits (e.g. Yang et al., 2011)—a multivariate accumulation of small influences. Consistent with this latter possibility, the *R*^2^ of all covariates combined was appreciable, even though very few individual predictors were significantly related to the cognitive changes. To detect specific effects that are individually small, but together combine to account for sizable proportions of variance, future research will likely require longitudinal samples that are many times larger than the one reported here.

## Figures and Tables

**Table 1:** Unstandardized means and variances for the intercept and slope of each cognitive test. Slopes refer to change from age 70 to age 76.

Cognitive domain	Cognitive test	Intercepts		Slopes		
		Mean (SE)	Variance (SE)	Mean (SE)	Variance (SE)	SD change/year
Visuospatial ability	Matrix Reasoning	13.451 (0.151)⁎⁎⁎	17.662 (1.248)⁎⁎⁎	− 0.156 (0.023)⁎⁎⁎	0.037 (0.044)	− 0.037
	Block Design	33.905 (0.307)⁎⁎⁎	83.340 (4.922)⁎⁎⁎	− 0.415 (0.038)⁎⁎⁎	0.168 (0.145)	− 0.046
	Spatial Span	7.359 (0.041)⁎⁎⁎	1.048 (0.097)⁎⁎⁎	− 0.027 (0.007)⁎⁎⁎	− 0.006 (0.004)	− 0.026
Crystallized ability	NART	34.365 (0.247)⁎⁎⁎	63.064 (2.853)⁎⁎⁎	− 0.026 (0.017)	0.070 (0.026)⁎⁎	− 0.003
	WTAR	40.992 (0.215)⁎⁎⁎	47.274 (2.189)⁎⁎⁎	− 0.079 (0.016)⁎⁎⁎	− 0.006 (0.023)	− 0.011
	Verbal Fluency	42.519 (0.378)⁎⁎⁎	135.289 (7.388)⁎⁎⁎	− 0.067 (0.046)	0.632 (0.218)⁎⁎	− 0.006
Verbal memory	Verbal Paired Associates	26.462 (0.280)⁎⁎⁎	66.140 (4.318)⁎⁎⁎	− 0.197 (0.043)⁎⁎⁎	0.435 (0.166)⁎	− 0.024
	Logical Memory	71.797 (0.537)⁎⁎⁎	244.534 (15.421)⁎⁎⁎	0.105 (0.089)	2.728 (0.585)⁎⁎⁎	0.007
	Digit Span Backward	7.749 (0.066)⁎⁎⁎	3.274 (0.247)⁎⁎⁎	− 0.023 (0.011)⁎	0.011 (0.010)	− 0.013
Processing speed	Symbol Search	24.680 (0.187)⁎⁎⁎	27.863 (1.846)⁎⁎⁎	− 0.149 (0.029)⁎⁎⁎	0.166 (0.063)⁎⁎	− 0.028
	Digit-Symbol Substitution	56.957 (0.388)⁎⁎⁎	139.946 (7.320)⁎⁎⁎	− 0.703 (0.047)⁎⁎⁎	0.443 (0.180)⁎	− 0.059
	Inspection Time	111.958 (0.333)⁎⁎⁎	79.257 (6.561)⁎⁎⁎	− 0.493 (0.065)⁎⁎⁎	0.899 (0.316)⁎⁎	− 0.055
	Choice Reaction Time	− 6.397 (0.026)⁎⁎⁎	0.547 (0.036)⁎⁎⁎	− 0.071 (0.004)⁎⁎⁎	0.001 (0.002)	− 0.096

Note: *p*-values uncorrected. All values from the baseline multivariate model in which all level and slope covariances were freely estimated. SE = standard error; SD change/year calculated by dividing the slope mean by the intercept standard deviation. NART = National Adult Reading Test; WTAR = Wechsler Test of Adult Reading. Choice Reaction Time was multiplied by − 10, such that higher scores indicated better performance.

⁎ *p* < 0.05.

⁎⁎ *p* < 0.01.

⁎⁎⁎ *p* < 0.001.

**Table 2:** Absolute and relative fit indices for the alternative structural models of cognitive level and slope.

Model number	Model description	*χ*^2^	df	RMSEA	CFI	TLI	SRMR	AIC	BIC	RMSEA comparator model	RMSEA of difference
1	Unstructured levels and Slopes	706.130	403	0.026	0.989	0.980	0.017	193,303.331	195,380.807	–	–
2	General factor of levels, unstructured slopes	3421.517	624	0.064	0.900	0.881	0.116	195,576.717	196,550.534	1	0.102
3	Hierarchical factor of levels, unstructured slopes	1479.931	620	0.036	0.969	0.963	0.057	193,643.132	194,636.924	1	0.049
4	Hierarchical levels, general slopes	1700.753	697	0.036	0.964	0.962	0.062	196,650.237	197,464.248	3	0.041
5	Fully hierarchical	1642.686	689	0.036	0.966	0.963	0.061	196,608.170	197,462.132	3	0.035

Note: RMSEA = Root Mean Square Error of Approximation; CFI = Comparative Fit Index; TLI = Tucker-Lewis Index; SRMR = Standardized Root Mean Square Residual; AIC = Akaike Information Criterion; BIC = Bayesian Information Criterion; ‘comparator model’ describes the model to which the RMSEA of difference column is relative.

**Table 3:** Associations of each predictor, all entered simultaneously, with the cognitive level and slope (cognitive ageing from 70 to 76) of cognitive ability from mean age. Table S7 (Supplemental materials) shows all effect sizes, and Table S4 shows the individual associations. Note that the general factor model and the domains model were run separately.

Covariate	General factor estimate (SE)		Domain estimate (SE)							
	*g* level	*g* slope	Visuospatial level	Crystallized level	Verbal memory level	Speed level	Visuospatial slope	Crystallized slope	Verbal memory slope	Speed slope
Age (baseline)	− 0.149 (0.043)⁎⁎	–	–	–	–	− 0.179 (0.058)⁎	0.394 (0.114)⁎	–	–	–
Sex (female)^a^	–	0.578 (0.192)⁎	–	–	–	0.409 (0.144)⁎	–	1.162 (0.295)⁎⁎	–	–
Time lag	–	–	–	–	–	–	–	–	–	–
Age 11 IQ	0.674 (0.031)⁎⁎⁎	–	0.461 (0.042)⁎⁎⁎	0.549 (0.031)⁎⁎	0.602 (0.052)⁎⁎⁎	0.432 (0.044)⁎⁎	–	–	–	–
Education	0.224 (0.036)⁎⁎	–	0.135 (0.047)⁎	0.250 (0.035)⁎⁎⁎	0.178 (0.055)⁎	–	–	–	–	–
Childhood SES	–	–	–	–	–	–	–	–	–	–
Own SES	–	–	–	–	–	–	–	–	–	–
SIMD	–	–	–	–	–	–	–	–	–	–
FEV	–	–	–	–	–	–	–	–	–	–
6 m walk time	–	–	–	–	–	–	–	–	–	–
Grip strength	–	0.262 (0.097)⁎	–	–	–	–	–	0.492 (0.150)⁎	–	–
*APOE*^a^	–	− 0.499 (0.114)⁎⁎	–	–	–	− 0.272 (0.088)⁎	–	–	− 0.357 (0.122)⁎	− 0.440 (0.127)⁎
BMI	–	–	0.132 (0.042)⁎	–	–	–	–	–	–	–
Smoking^a^	–	–	–	–	–	–	–	–	–	–
Alcohol	–	–	–	–	–	–	–	–	–	–
CVD^a^	–	–	–	–	–	–	–	–	–	–
Hypertension^a^	–	–	–	–	–	–	–	–	–	–
Diabetes^a^	–	–	–	–	–	–	–	–	–	–

Note: All *p*-values corrected for False Discovery Rate. *g* = general factor; age 11 IQ = cognitive ability assessed by the Moray House Test No. 12; SES = occupational socioeconomic status; SIMD = Scottish Index of Multiple Deprivation; FEV = Forced Expiratory Volume in 1 s; BMI = Body Mass Index; CVD = Cardiovascular Disease history. Cells with dashes represent non-significant effects (note some predictors show no associations with any cognitive factors). All factors were estimated within the hierarchical model.

⁎ *p* < 0.05.

⁎⁎ *p* < 0.01.

⁎⁎⁎ *p* < 0.001.

a Categorical covariate; effect sizes expressed in terms of Cohen's *d; other effects are standardized betas*.

## Appendix A Supplementary data

Supplementary Method, Supplementary Results, Tables S1-S7, Figure S1. Image 1
