# Supplementary material for: Predictors of ageing-related decline across multiple cognitive functions
Source: Intelligence. 2016 Nov-Dec;59:115–26. doi: 10.1016/j.intell.2016.08.007 (PMC5127886; doi:10.1016/j.intell.2016.08.007)
Supplement: Supplementary file 1 — Supplementary Method, Supplementary Results, Tables S1-S7, Figure S1. [file mmc1.docx]

**Supplementary Materials for:**

**Predictors of ageing-related decline across multiple cognitive functions**

Stuart J. Ritchie*, Elliot M. Tucker-Drob, Simon R. Cox, Janie Corley, Dominika Dykiert, Paul Redmond, Alison Pattie, Adele M. Taylor, Ruth Sibbett, John M. Starr, & Ian J. Deary

*To whom correspondence should be addressed:

stuart.ritchie@ed.ac.uk

**Supplementary Method**

**Structure of cognitive change**

To calculate the amount of variance explained at each level of the model, we used the following process for both levels and slopes. For each test, to calculate the proportion of variance due to the general factor, we multiplied the standardized loading of the test on its domain (see Fig. 3 in the main article) by the loading of that domain on the general factor, squaring the result. To calculate the variance explained by the domain, we squared the loading of the test on its domain, then subtracted the proportion of variance at the general-factor level from the result. To calculate the variance explained at the individual test level, we subtracted both numbers from 1. Averaging the general, domain, and test-specific values across all thirteen tests, and converting the proportion results into percentages, gave us the percentage variance results described in the main document.

**Supplementary Results**

**Alternative structural models**

For the study’s main analysis, we used a hierarchical model (e.g. Carroll, 1993) with a *g*-factor superimposed on four domain factors on the theoretical basis outlined by Tucker-Drob et al. (2014). Homologous versions of this hierarchical factor model were implemented for the structure of the levels and structure of the slopes. For completeness, we also tested two alternative models for the structure of the slopes. These models used as their basis the theoretical dichotomy of cognitive ‘mechanics’ and ‘pragmatics’ (*a la* Baltes, 1993). The first alternative model had two domain factors for both level and slope (in addition to the higher-order general factors of level and slope). The ‘mechanics’ domain factor was a combination of all the tests involved in the Visuospatial, Verbal Memory, and Speed tasks in the main analysis (i.e. Matrix Reasoning, Block Design, Spatial Span, Verbal Paired Associates, Logical Memory, Digit Span Backward, Symbol Search, Digit-Symbol Substitution, Inspection Time, and Reaction Time) and the ‘pragmatics’ domain factor included identical tests to the Crystallized domain in the main analysis (i.e. NART, WTAR, and Verbal Fluency). This model had good fit to the data (*χ*^2^(701) = 2228.96, *p* < .001, RMSEA = .045, CFI = .945, TLI = .942). However, the fit was significantly poorer than the four-domain factor model from the main document (*χ*^2^(12) = 586.28, *p* < .001; ΔAIC = 562.28, ΔBIC = 502.35).

The second alternative model retained the four-domain-factor structure from the main analysis for level structure (i.e. factors of Visuospatial ability, Crystallized ability, Verbal Memory, and Speed), but used the ‘mechanics’ and ‘pragmatics’ domain factors, as above, for the slope structure. Again, this model fit the data well (*χ*^2^(701) = 1689.69, *p* < .001, RMSEA = .036, CFI = .964, TLI = .962). The fit was also significantly poorer than the model with four domains for both levels and slopes (*χ*^2^(4) = 47.00, *p* < .001; ΔAIC = 39.00, ΔBIC = 19.03). Overall, comparison to other theoretically-plausible models (which were both well-fitting in an absolute sense) showed that our four-factor model had the better fit to the data.

**Sensitivity Analyses**

Four sensitivity analyses were performed. First, it has been suggested that the effects of *APOE* e4 are mainly due to dementia pathology, and are thus not present in non-pathological cognitive ageing (Bunce et al., 2014). For this reason, we ran a sensitivity analysis where we removed all individuals who scored lower than 24 (a commonly-used cutoff indicating possible dementia) on the Mini Mental State Examination (MMSE; Folstein, Folstein, & McHugh, 1975) at any of the three testing waves. This involved a reduction in the total valid sample of 13 participants. In the fully-adjusted model of general factors, there remained a significant association of e4 status with the general slope factor (*d* = −0.525, *p* = .001). In the domains model, the effect sizes were only slightly smaller than in the main model: the association of e4 status with Speed remained significant (*d* = −0.423, *p* = .028), though that with Verbal Memory dropped below the *p* < .05 significance threshold after False Discovery Rate correction (*d* = −0.285, *p* = .13).

Second, for the same reasons as the MMSE exclusion analyses, we ran a further sensitivity analysis excluding 25 individuals who prospectively were classified as having dementia; no LBC1936 participants had dementia at the first wave of testing at age 70. This was done either by inspection of their death certificate, their health status from the Scottish Morbidity Records (Information Services Division Scotland, http://www.isdscotland.org/index.asp), a dataset updated on the basis of hospital admission records, inpatient psychiatric hospital records, and cancer registrations, or by clinical reviews carried out by clinicians working in tandem with the Lothian Birth Cohort study. All records were coded using the International Classification of Disease versions 9 or 10 (ICD-9 or ICD-10). In the model excluding these individuals, the relation of *APOE* e4 status to the general change factor was still present (*d* = −0.418, *p* = .007). As with the above analysis, in the domains model there was still an association of e4 status with change in Speed (*d* = −0.380, *p* = .038), though not with Verbal Memory (*d* = −0.305, *p* = .100). With some exceptions, these two sensitivity analyses indicated that the associations we found between *APOE* e4 and cognitive ageing were generally still present in those with no indication—either behavioural or diagnostic—of dementia symptoms.

Third, since participant mood state could potentially affect the results, we ran a sensitivity model including Hospital Anxiety and Depression Scale (HADS; Zigmond & Snaith, 1983) score as a time-varying covariate in the fully-adjusted model. That is, we regressed each test at each testing wave on the HADS total score measured at that age, thereby adjusting each score for any association with anxiety and depression mood states. Of the 39 associations (one for each test at each wave), 18 were statistically significant, and all were negative (higher anxiety/depression scores were related to lower cognitive abilities) but none had an absolute standardized effect size larger than .097. Including mood in the model in this way did not substantially alter any of the significant or non-significant results shown in Table S7.

The fourth sensitivity analysis, which tested whether different predictions were made when including a general factor of physical fitness instead of separate grip strength, lung function and walking speed predictors, is detailed in the main document.

**Supplementary References**

Baltes, P.B. (1993). The aging mind: Potential and limits. *The Gerontologist*, **33** (5), 1993, 580-594, doi: http://dx.doi.org/10.1093/geront/33.5.580

Bunce, D., Bielak, A.A., Anstey, K.J., Cherbuin, N., Batterham, P.J., & Easteal, S. (2014). *APOE* genotype and cognitive change in young, middle-aged, and older adults living in the community. *Journals of Gerontology A: Biological Sciences and Medical Sciences*, **69**, 2014, 379-86, http://dx.doi.org/10.1093/gerona/glt103.

Carroll, J. B. (1993). *Human cognitive abilities: A survey of factor-analytic studies*. Cambridge, England: Cambridge University Press.

Folstein, M.F., Folstein, S.E., & McHugh, P.R. “Mini Mental State”: a practical method for grading the cognitive state of patients for the clinician. *Journal of Psychiatric Research* **12**, 1975, 189-198, http://dx.doi.org/10.1016/0022-3956(75)90026-6.

Tucker-Drob, E.M., Briley, D.A., Starr, J.M., & Deary, I.J. Structure and correlates of cognitive aging in a narrow age cohort. *Psychology and Aging*, **29**, 2014, 236-249, http://dx.doi.org/10.1037/a0036187.

Zigmond, A.S., & Snaith, R.P. The hospital anxiety and depression scale. *Acta Psychiatrica Scandinavia* **67**, 1983, 361-370, http://dx.doi.org/10.1111/j.1600-0447.1983.tb09716.x.

**Supplementary Tables**

*Table S1.* Descriptive statistics for each cognitive test and covariate at each testing wave.

|  | Variable | Wave 1 | | | Wave 2 | | | Wave 3 | | |
| --- | --- | --- | --- | --- | --- | --- | --- | --- | --- | --- |
| Cognitive Tests |  | *n* | M | SD | *n* | M | SD | *n* | M | SD |
|  | Matrix Reasoning | 1086 | 13.49 | 5.13 | 863 | 13.17 | 4.96 | 689 | 13.04 | 4.91 |
|  | Block Design | 1085 | 33.79 | 10.32 | 864 | 33.64 | 10.08 | 691 | 32.18 | 9.95 |
|  | Spatial Span | 1084 | 7.36 | 1.42 | 861 | 7.35 | 1.38 | 690 | 7.31 | 1.36 |
|  | NART | 1089 | 34.48 | 8.16 | 864 | 34.38 | 8.18 | 695 | 35.00 | 8.05 |
|  | WTAR | 1089 | 41.02 | 7.18 | 864 | 41.01 | 6.97 | 694 | 41.09 | 7.03 |
|  | Verbal Fluency | 1087 | 42.42 | 12.54 | 865 | 43.18 | 12.94 | 696 | 42.90 | 12.77 |
|  | Verbal Paired Associates | 1050 | 26.44 | 9.13 | 843 | 27.18 | 9.46 | 663 | 26.41 | 9.56 |
|  | Logical Memory | 1087 | 71.37 | 17.95 | 864 | 74.23 | 17.89 | 688 | 74.58 | 19.20 |
|  | Digit Span Backward | 1090 | 7.73 | 2.26 | 866 | 7.81 | 2.29 | 695 | 7.77 | 2.37 |
|  | Symbol Search | 1086 | 24.71 | 6.39 | 862 | 24.61 | 6.18 | 687 | 24.60 | 6.46 |
|  | Digit-Symbol | 1086 | 56.60 | 12.93 | 862 | 56.40 | 12.31 | 685 | 53.81 | 12.93 |
|  | Inspection Time | 1041 | 112.14 | 11.00 | 838 | 111.22 | 11.79 | 654 | 110.17 | 12.53 |
|  | Choice Reaction Time | 1084 | 0.64 | 0.09 | 865 | 0.65 | 0.09 | 685 | 0.68 | 0.10 |
| Covariates | Age 11 IQ | 1028 | 40.00 | 11.80 | - | - | - | - | - | - |
|  | Education | 1091 | 10.74 | 1.13 | - | - | - | - | - | - |
|  | Childhood SES | 960 | 2.91 | .940 | - | - | - | - | - | - |
|  | Own SES | 1070 | 2.59 | 1.16 | - | - | - | - | - | - |
|  | SIMD | 1083 | 6.25 | 2.09 | - | - | - | - | - | - |
|  | FEV | 1085 | 2.36 | .69 | - | - | - | - | - | - |
|  | 6m Walk Time | 1085 | 3.86 | 1.16 | - | - | - | - | - | - |
|  | Grip Strength | 1083 | 28.03 | 9.92 | - | - | - | - | - | - |
|  | BMI | 1089 | 27.78 | 4.36 | - | - | - | - | - | - |
|  | Alcohol g per week | 928 | 11.98 | 16.79 |  |  |  |  |  |  |
|  | HADS (time-varying) | 1086 | 7.68 | 4.52 | 865 | 7.14 | 4.45 | 696 | 7.57 | 4.53 |
| Covariates (dichotomous) |  | *n*  total | *n* present | *n*  absent |  |  |  |  |  |  |
|  | APOE ε4 allele | 1028 | 306 | 722 | - | - | - | - | - | - |
|  | Smoker/ex-smoker | 1091 | 590 | 501 | - | - | - | - | - | - |
|  | Cardiovascular disease | 1091 | 268 | 823 | - | - | - | - | - | - |
|  | Hypertension | 1091 | 433 | 658 | - | - | - | - | - | - |
|  | Diabetes | 1091 | 91 | 1000 | - | - | - | - | - | - |

Note: Wave 1: mean age 70 years; wave 2: mean age 73 years; wave 3: mean age 76 years. NART = National Adult Reading Test; WTAR = Wechsler Test of Adult Reading; SES = Socioeconomic Status; SIMD = Scottish Index of Multiple Deprivation; FEV = forced expiratory volume in 1 second; BMI = body mass index; HADS = Hospital Anxiety and Depression Scale total score. The means here are raw and not FIML-estimated.

*Table S2.* Pearson correlation matrix for each cognitive test at each testing wave.

| Variable | 1. | 2. | 3. | 4. | 5. | 6. | 7. | 8. | 9. | 10. | 11. | 12. | 13. | 14. | 15. | 16. | 17. | 18. | 19. | 20. | 21. | 22. | 23. | 24. | 25. | 26. | 27. | 28. | 29. | 30. | 31. | 32. | 33. | 34. | 35. | 36. | 37. | 38. |
| --- | --- | --- | --- | --- | --- | --- | --- | --- | --- | --- | --- | --- | --- | --- | --- | --- | --- | --- | --- | --- | --- | --- | --- | --- | --- | --- | --- | --- | --- | --- | --- | --- | --- | --- | --- | --- | --- | --- |
| 1. Matrix reasoning 1 | - |  |  |  |  |  |  |  |  |  |  |  |  |  |  |  |  |  |  |  |  |  |  |  |  |  |  |  |  |  |  |  |  |  |  |  |  |  |
| 2. Matrix reasoning 2 | .65 | - |  |  |  |  |  |  |  |  |  |  |  |  |  |  |  |  |  |  |  |  |  |  |  |  |  |  |  |  |  |  |  |  |  |  |  |  |
| 3. Matrix reasoning 3 | .63 | .64 | - |  |  |  |  |  |  |  |  |  |  |  |  |  |  |  |  |  |  |  |  |  |  |  |  |  |  |  |  |  |  |  |  |  |  |  |
| 4. Block design 1 | .57 | .55 | .56 | - |  |  |  |  |  |  |  |  |  |  |  |  |  |  |  |  |  |  |  |  |  |  |  |  |  |  |  |  |  |  |  |  |  |  |
| 5. Block design 2 | .52 | .53 | .51 | .76 | - |  |  |  |  |  |  |  |  |  |  |  |  |  |  |  |  |  |  |  |  |  |  |  |  |  |  |  |  |  |  |  |  |  |
| 6. Block design 3 | .55 | .54 | .57 | .76 | .76 | - |  |  |  |  |  |  |  |  |  |  |  |  |  |  |  |  |  |  |  |  |  |  |  |  |  |  |  |  |  |  |  |  |
| 7. Spatial Span 1 | .38 | .35 | .37 | .40 | .40 | .41 | - |  |  |  |  |  |  |  |  |  |  |  |  |  |  |  |  |  |  |  |  |  |  |  |  |  |  |  |  |  |  |  |
| 8. Spatial Span 2 | .37 | .36 | .38 | .42 | .45 | .44 | .56 | - |  |  |  |  |  |  |  |  |  |  |  |  |  |  |  |  |  |  |  |  |  |  |  |  |  |  |  |  |  |  |
| 9. Spatial Span 3 | .30 | .34 | .41 | .36 | .37 | .44 | .58 | .57 | - |  |  |  |  |  |  |  |  |  |  |  |  |  |  |  |  |  |  |  |  |  |  |  |  |  |  |  |  |  |
| 10. NART 1 | .45 | .40 | .42 | .41 | .37 | .39 | .22 | .19 | .16 | - |  |  |  |  |  |  |  |  |  |  |  |  |  |  |  |  |  |  |  |  |  |  |  |  |  |  |  |  |
| 11. NART 2 | .42 | .39 | .41 | .39 | .36 | .37 | .21 | .18 | .17 | .94 | - |  |  |  |  |  |  |  |  |  |  |  |  |  |  |  |  |  |  |  |  |  |  |  |  |  |  |  |
| 12. NART 3 | .42 | .40 | .43 | .41 | .37 | .40 | .19 | .17 | .18 | .92 | .94 | - |  |  |  |  |  |  |  |  |  |  |  |  |  |  |  |  |  |  |  |  |  |  |  |  |  |  |
| 13. WTAR 1 | .44 | .37 | .39 | .39 | .35 | .38 | .25 | .18 | .18 | .89 | .89 | .88 | - |  |  |  |  |  |  |  |  |  |  |  |  |  |  |  |  |  |  |  |  |  |  |  |  |  |
| 14. WTAR 2 | .42 | .40 | .40 | .40 | .37 | .38 | .23 | .19 | .18 | .89 | .91 | .89 | .93 | - |  |  |  |  |  |  |  |  |  |  |  |  |  |  |  |  |  |  |  |  |  |  |  |  |
| 15. WTAR 3 | .42 | .39 | .40 | .37 | .33 | .36 | .19 | .14 | .17 | .87 | .89 | .89 | .92 | .92 | - |  |  |  |  |  |  |  |  |  |  |  |  |  |  |  |  |  |  |  |  |  |  |  |
| 16. Verbal Fluency 1 | .29 | .22 | .23 | .26 | .22 | .22 | .17 | .16 | .13 | .47 | .45 | .42 | .47 | .46 | .42 | - |  |  |  |  |  |  |  |  |  |  |  |  |  |  |  |  |  |  |  |  |  |  |
| 17. Verbal Fluency 2 | .26 | .21 | .24 | .25 | .23 | .22 | .15 | .19 | .14 | .45 | .45 | .44 | .44 | .45 | .43 | .81 | - |  |  |  |  |  |  |  |  |  |  |  |  |  |  |  |  |  |  |  |  |  |
| 18. Verbal Fluency 3 | .28 | .20 | .25 | .23 | .25 | .24 | .13 | .15 | .18 | .45 | .45 | .44 | .45 | .45 | .44 | .78 | .82 | - |  |  |  |  |  |  |  |  |  |  |  |  |  |  |  |  |  |  |  |  |
| 19. Paired Assoc. 1 | .31 | .27 | .30 | .27 | .22 | .19 | .16 | .11 | .11 | .37 | .38 | .38 | .37 | .37 | .35 | .25 | .24 | .23 | - |  |  |  |  |  |  |  |  |  |  |  |  |  |  |  |  |  |  |  |
| 20. Paired Assoc. 2 | .32 | .29 | .28 | .26 | .24 | .18 | .13 | .18 | .15 | .37 | .38 | .37 | .37 | .37 | .36 | .27 | .32 | .28 | .72 | - |  |  |  |  |  |  |  |  |  |  |  |  |  |  |  |  |  |  |
| 21. Paired Assoc. 3 | .26 | .27 | .30 | .20 | .16 | .19 | .12 | .10 | .17 | .38 | .41 | .41 | .39 | .40 | .39 | .25 | .32 | .34 | .67 | .70 | - |  |  |  |  |  |  |  |  |  |  |  |  |  |  |  |  |  |
| 22. Logical Mem. 1 | .33 | .30 | .29 | .28 | .23 | .23 | .24 | .17 | .15 | .45 | .45 | .43 | .45 | .41 | .41 | .23 | .23 | .18 | .48 | .38 | .36 | - |  |  |  |  |  |  |  |  |  |  |  |  |  |  |  |  |
| 23. Logical Mem. 2 | .33 | .33 | .29 | .28 | .28 | .26 | .21 | .23 | .22 | .42 | .43 | .45 | .41 | .41 | .43 | .21 | .25 | .23 | .43 | .52 | .45 | .70 | - |  |  |  |  |  |  |  |  |  |  |  |  |  |  |  |
| 24. Logical Mem. 3 | .32 | .30 | .32 | .24 | .23 | .30 | .20 | .19 | .25 | .39 | .42 | .43 | .40 | .39 | .40 | .16 | .21 | .25 | .40 | .40 | .53 | .61 | .74 | - |  |  |  |  |  |  |  |  |  |  |  |  |  |  |
| 25. Digit Span 1 | .40 | .34 | .37 | .34 | .32 | .34 | .32 | .31 | .31 | .43 | .43 | .45 | .44 | .42 | .43 | .31 | .32 | .30 | .27 | .25 | .24 | .30 | .30 | .26 | - |  |  |  |  |  |  |  |  |  |  |  |  |  |
| 26. Digit Span 2 | .34 | .35 | .34 | .31 | .29 | .29 | .30 | .31 | .30 | .38 | .39 | .40 | .38 | .41 | .39 | .30 | .32 | .31 | .24 | .27 | .28 | .28 | .31 | .29 | .64 | - |  |  |  |  |  |  |  |  |  |  |  |  |
| 27. Digit Span 3 | .39 | .35 | .38 | .30 | .28 | .32 | .31 | .32 | .31 | .39 | .41 | .41 | .42 | .44 | .43 | .32 | .32 | .34 | .25 | .26 | .31 | .28 | .28 | .32 | .65 | .68 | - |  |  |  |  |  |  |  |  |  |  |  |
| 28. Symbol Search 1 | .45 | .38 | .39 | .48 | .46 | .50 | .41 | .39 | .31 | .40 | .39 | .40 | .41 | .40 | .38 | .37 | .36 | .37 | .22 | .22 | .20 | .33 | .30 | .29 | .34 | .34 | .32 | - |  |  |  |  |  |  |  |  |  |  |
| 29. Symbol Search 2 | .40 | .38 | .38 | .47 | .48 | .50 | .38 | .39 | .36 | .36 | .37 | .38 | .35 | .39 | .34 | .29 | .33 | .31 | .19 | .21 | .19 | .28 | .29 | .28 | .30 | .33 | .29 | .67 | - |  |  |  |  |  |  |  |  |  |
| 30. Symbol Search 3 | .38 | .40 | .44 | .44 | .47 | .53 | .34 | .42 | .40 | .34 | .35 | .36 | .35 | .36 | .35 | .24 | .29 | .33 | .19 | .27 | .26 | .24 | .31 | .36 | .32 | .35 | .35 | .63 | .67 | - |  |  |  |  |  |  |  |  |
| 31. Digit Symbol 1 | .37 | .34 | .34 | .39 | .37 | .37 | .31 | .30 | .25 | .40 | .40 | .40 | .38 | .40 | .38 | .36 | .35 | .36 | .24 | .24 | .25 | .31 | .30 | .30 | .30 | .31 | .29 | .62 | .58 | .58 | - |  |  |  |  |  |  |  |
| 32. Digit Symbol 2 | .36 | .38 | .36 | .40 | .40 | .41 | .31 | .32 | .29 | .41 | .42 | .44 | .39 | .42 | .41 | .34 | .38 | .37 | .24 | .26 | .28 | .29 | .33 | .35 | .33 | .33 | .34 | .59 | .63 | .62 | .85 | - |  |  |  |  |  |  |
| 33. Digit Symbol 3 | .34 | .36 | .40 | .37 | .35 | .44 | .27 | .27 | .32 | .36 | .36 | .39 | .36 | .37 | .36 | .30 | .33 | .40 | .20 | .21 | .28 | .27 | .32 | .40 | .30 | .31 | .34 | .56 | .58 | .65 | .77 | .83 | - |  |  |  |  |  |
| 34. Inspection Time 1 | .21 | .17 | .15 | .27 | .23 | .27 | .27 | .26 | .22 | .13 | .08 | .10 | .15 | .09 | .07 | .17 | .18 | .16 | .09 | .12 | .08 | .07 | .10 | .08 | .17 | .15 | .15 | .32 | .28 | .26 | .30 | .31 | .30 | - |  |  |  |  |
| 35. Inspection Time 2 | .27 | .26 | .25 | .29 | .30 | .33 | .26 | .29 | .24 | .21 | .23 | .23 | .21 | .21 | .19 | .23 | .26 | .23 | .14 | .19 | .17 | .10 | .19 | .18 | .17 | .19 | .18 | .35 | .36 | .34 | .31 | .38 | .32 | .59 | - |  |  |  |
| 36. Inspection Time 3 | .27 | .26 | .29 | .25 | .26 | .35 | .24 | .27 | .26 | .20 | .20 | .19 | .21 | .20 | .20 | .17 | .21 | .25 | .09 | .14 | .18 | .12 | .14 | .19 | .15 | .13 | .18 | .34 | .34 | .41 | .28 | .34 | .37 | .51 | .59 | - |  |  |
| 37. Reaction Time 1 | .27 | .23 | .23 | .32 | .28 | .28 | .36 | .33 | .31 | .25 | .25 | .21 | .24 | .23 | .18 | .24 | .26 | .26 | .17 | .16 | .18 | .23 | .19 | .20 | .25 | .21 | .23 | .48 | .47 | .44 | .51 | .53 | .49 | .36 | .34 | .28 | - |  |
| 38. Reaction Time 2 | .28 | .25 | .24 | .32 | .32 | .32 | .31 | .35 | .31 | .24 | .24 | .24 | .21 | .23 | .22 | .23 | .30 | .30 | .14 | .22 | .21 | .18 | .27 | .24 | .26 | .22 | .21 | .45 | .50 | .50 | .52 | .55 | .53 | .36 | .38 | .31 | .75 | - |
| 39. Reaction Time 3 | .26 | .25 | .28 | .28 | .26 | .36 | .29 | .32 | .35 | .22 | .20 | .21 | .20 | .20 | .17 | .15 | .23 | .30 | .12 | .18 | .25 | .12 | .19 | .29 | .23 | .20 | .26 | .40 | .40 | .53 | .44 | .48 | .56 | .32 | .33 | .36 | .67 | .71 |

Note: Numbers 1/2/3 refer to testing waves 1, 2, and 3 respectively. NART = National Adult Reading Test; WTAR = Wechsler Test of Adult Reading; Paired Assoc. = Verbal Paired Associates; Logical Mem. = Logical Memory; Digit Span = Digit Span Backward.

*Table S3.* Heterogeneous correlation matrix for each covariate.

| Variable | 1. | 2. | 3. | 4. | 5. | 6. | 7. | 8. | 9. | 10. | 11. | 12. | 13. | 14. | 15. | 16. | 17. |
| --- | --- | --- | --- | --- | --- | --- | --- | --- | --- | --- | --- | --- | --- | --- | --- | --- | --- |
| 1. Age 11 IQ | - |  |  |  |  |  |  |  |  |  |  |  |  |  |  |  |  |
| 2. Education | .412 | - |  |  |  |  |  |  |  |  |  |  |  |  |  |  |  |
| 3. Origin SES | −.199 | −.337 | - |  |  |  |  |  |  |  |  |  |  |  |  |  |  |
| 4. Own SES | −.338 | −.431 | .236 | - |  |  |  |  |  |  |  |  |  |  |  |  |  |
| 5. SIMD | .297 | .339 | −.230 | −.354 | - |  |  |  |  |  |  |  |  |  |  |  |  |
| 6. FEV | .017 | .088 | −.096 | −.042 | .229 | - |  |  |  |  |  |  |  |  |  |  |  |
| 7. 6m Walk time | .167 | .171 | −.135 | −.093 | .180 | .252 | - |  |  |  |  |  |  |  |  |  |  |
| 8. Grip strength | −.044 | .054 | −.114 | .050 | .057 | .648 | .285 | - |  |  |  |  |  |  |  |  |  |
| 9. BMI | −.135 | −.123 | .022 | .103 | −.145 | −.060 | −.199 | .037 | - |  |  |  |  |  |  |  |  |
| 10. HADS wave 1 | −.147 | −.080 | .007 | .070 | −.043 | −.131 | −.126 | −.153 | −.021 | - |  |  |  |  |  |  |  |
| 11. HADS wave 2 | −.114 | −.059 | .019 | .045 | −.060 | −.172 | −.116 | −.148 | −.018 | .740 | - |  |  |  |  |  |  |
| 12. HADS wave 3 | −.160 | −.086 | .011 | .086 | −.071 | −.162 | −.111 | −.154 | −.031 | .631 | .735 | - |  |  |  |  |  |
| 13. *APOE* | −.005 | −.034 | .062 | .020 | −.015 | .085 | .027 | .025 | −.009 | −.044 | −.085 | −.032 | - |  |  |  |  |
| 14. Smoking status | −.099 | −.103 | .023 | −.076 | −.203 | −.064 | .080 | .148 | .091 | .056 | .067 | .052 | −.084 | - |  |  |  |
| 15. Alcohol | .146 | .115 | −.131 | −.171 | .187 | .284 | −.205 | .361 | −.002 | 0.144 | −.136 | -.101 | .015 | .108 | - |  |  |
| 16. CVD | −.208 | −.119 | .103 | .036 | −.175 | −.067 | −.170 | .024 | .145 | .052 | .089 | .072 | .033 | .178 | −.001 | - |  |
| 17. Hypertension | .015 | −.045 | .032 | −.032 | −.036 | −.140 | −.183 | −.132 | .244 | .055 | .132 | .143 | −.068 | −.072 | −.097 | 0.398 | - |
| 18. Diabetes | −.171 | −.134 | .041 | .038 | −.160 | −.045 | −.101 | −.057 | .253 | .106 | .132 | .183 | −.021 | .158 | −.132 | 0.362 | .451 |

Note: The heterogeneous correlation matrix includes Pearson correlations between continuous variables, polyserial correlations between continuous and ordinal/categorical variables, and polychoric/tetrachoric correlations between ordinal/categorical variables. IQ = cognitive ability; SES = socioeconomic status; SIMD = Scottish Index of Multiple Deprivation; FEV = forced expiratory volume; BMI = body mass index; HADS = Hospital Anxiety and Depression Scale, total score; *APOE* = *APOE* e4 carrier; Alcohol = alcohol g consumed per week; CVD = cardiovascular disease.

*Table S4*. Standardized associations of each predictor, entered individually alongside age and sex, on the level and slope of decline in the general factors of level and slope and—in a separate model—each of the four cognitive domains.

| Covariate | General factor estimate (SE) | |  | Domain factor estimate (SE) | | | | | | | |
| --- | --- | --- | --- | --- | --- | --- | --- | --- | --- | --- | --- |
|  | *g* level | *g* slope |  | Visuospatial  level | Crystallized  level | Verbal memory  level | Speed  level | Visuospatial  slope | Crystallized  slope | Verbal memory  slope | Speed  slope |
| Age (baseline) | −.728 (.106)^***^ | .021 (.007)^**^ |  | −.209 (.032)^***^ | −.167 (.030)^***^ | −.245 (.035)^***^ | −.225 (.032)^***^ | .383 (.074)^***^ | .108 (.071) | .181 (.048)^***^ | .047 (.050) |
| Sex (female) † | −.009 (.068) | .135 (.096) |  | −.411 (.065)^***^ | .136 (.061)^*^ | .267 (.073)^***^ | .092 (.066) | .122 (.145) | .260 (.140) | .085 (.098) | .035 (.100) |
| Time lag | −.033 (.054) | .015 (.065) |  | −.001 (.054) | −.020 (.049) | −.046 (.061) | −.056 (.053) | .089 (.096) | .037 (.098) | .036 (.066) | −.036 (.069) |
| Age 11 IQ | .786 (.017)^***^ | −.143 (.053)^**^ |  | .586 (.025)^***^ | .702 (.017)^***^ | .637 (.029)^***^ | .533 (.025)^***^ | −.170 (.071)^*^ | .004 (.073) | −.035 (.049) | −.079 (.051) |
| Education | .530 (.026)^***^ | −.095 (.046)^*^ |  | .385 (.029)^***^ | .537 (.022)^***^ | .401 (.032)^***^ | .309 (.030)^***^ | −.070 (.074) | −.171 (.068)^*^ | −.022 (.048) | −.017 (.049) |
| Childhood SES | .258 (.034)^***^ | −.067 (.049) |  | .165 (.035)^***^ | .292 (.030)^***^ | .135 (.039)^**^ | .208 (.034)^***^ | .021 (.075) | −.138 (.076) | −.048 (.050) | −.018 (.052) |
| Own SES | .360 (.030)^***^ | −.057 (.046) |  | .309 (.031)^***^ | .362 (.027)^***^ | .234 (.036)^***^ | .221 (.032)^***^ | −.017 (.072) | −.087 (.067) | −.007 (.049) | −.028 (.049) |
| SIMD | .373 (.030)^***^ | −.030 (.050) |  | .299 (.032)^***^ | .324 (.028)^***^ | .253 (.036)^***^ | .310 (.031)^***^ | −.020 (.076) | −.044 (.072) | −.057 (.052) | .026 (.053) |
| FEV | .324 (.040)^***^ | .167 (.060)^**^ |  | .284 (.040)^***^ | .235 (.037)^***^ | .148 (.046)^**^ | .340 (.039)^***^ | .206 (.092)^*^ | .154 (.089) | .086 (.062) | .134 (.063)^*^ |
| 6m Walk time | .276 (.032)^***^ | .132 (.053)^*^ |  | .228 (.033)^***^ | .205 (.030)^***^ | .155 (.037)^***^ | .276 (.032)^***^ | .165 (.082)^*^ | .009 (.078) | .064 (.056) | .157 (.057)^**^ |
| Grip strength | .391 (.051)^***^ | .330 (.077)^***^ |  | .391 (.052)^***^ | .285 (.049)^***^ | .205 (.060)^**^ | .352 (.051)^***^ | .169 (.120) | .425 (.117)^***^ | .240 (.081)^**^ | .246 (.083)^**^ |
| *APOE*† | −.132 (.074) | −.395 (.101)^***^ |  | −.170 (.075)^*^ | .021 (.069) | −.083 (.082) | −.167 (.073)^*^ | −.334 (.157)^*^ | .049 (.154) | −.327 (.105)^**^ | −.387 (.108)^***^ |
| BMI | −.111 (.033)^**^ | −.055 (.047) |  | −.054 (.034) | −.187 (.030)^***^ | −.043 (.037) | −.068 (.033)^*^ | .006 (.073) | −.012 (.069) | −.083 (.049) | −.074 (.050) |
| Smoking† | −.186 (.066)^**^ | −.113 (.093) |  | −.224 (.067)^***^ | −.077 (.062) | −.022 (.074) | −.235 (.066)^***^ | −.161 (.143) | −.076 (.142) | −.104 (.096) | −.045 (.099) |
| Alcohol | .246 (.037)^***^ | −.034 (.053) |  | .203 (.038)^***^ | .216 (.034)^***^ | .205 (.043)^***^ | .160 (.038)^***^ | −.185 (.078)^*^ | .081 (.077) | −.014 (.055) | −.014 (.057) |
| CVD† | −.187 (.077)^*^ | −.163 (.110) |  | −.195 (.078)^*^ | −.086 (.071) | .002 (.086) | −.294 (.075)^***^ | −.053 (.118) | −.367 (.163)^*^ | −.091 (.115) | −.053 (.118) |
| Hypertension† | −.176 (.067)^**^ | .020 (.097) |  | −.151 (.068)^*^ | −.134 (.062)^*^ | −.083 (.075) | −.175 (.066)^**^ | .035 (.147) | .119 (.140) | .086 (.099) | −.075 (.102) |
| Diabetes† | −.486 (.117)^***^ | −.310 (.171) |  | −.434 (.119)^***^ | −.456 (.109)^***^ | −.211 (.132) | −.446 (.117)^***^ | −.072 (.267) | −.384 (.254) | −.258 (.180) | −.267 (.185) |

Note: * = *p* < .05; ** = *p* <.01; *** = *p* < .001; *p*-values uncorrected. † = categorical predictor; all other predictors continuous. Continuous predictors standardized with respect to both the predictor and the outcome; categorical predictors standardized with respect to the outcome only (analogous to Cohen’s *d*). *g* = general factor; SES = Socioeconomic Status; SIMD = Scottish Index of Multiple Deprivation; FEV = Forced Expiratory Volume in 1 second; *APOE* = *APOE* e4 carrier; Alcohol = alcohol g consumed per week; CVD = Cardiovascular Disease.

*Table S5*. Standardized associations of each predictor, entered individually alongside age, sex, and age 11 IQ, on the level of the general factors and—in a separate model—the level in each of the four cognitive domains.

| Covariate | General factor estimate (SE) |  | Domain factor estimate (SE) | | | |
| --- | --- | --- | --- | --- | --- | --- |
|  | *g* level |  | Visuospatial  level | Crystallized  level | Verbal memory  level | Speed  level |
| Age (baseline) | −.223 (.023)^***^ |  | −.193 (.029)^***^ | −.138 (.023)^***^ | −.230 (.032)^***^ | −.210 (.029)^***^ |
| Sex (female) † | −.041 (.048) |  | −.482 (.057)^***^ | .056 (.045) | .186 (.065)^**^ | .042 (.060) |
| Time lag | −.056 (.037) |  | −.007 (.048) | −.066 (.037) | −.049 (.054) | −.039 (.048) |
| Age 11 IQ | .786 (.017)^***^ |  | .586 (.025)^***^ | .702 (.017)^***^ | .637 (.029)^***^ | .533 (.025)^***^ |
| Education | .255 (.024)^***^ |  | .166 (.032)^***^ | .284 (.023)^***^ | .152 (.035)^***^ | .096 (.032)^**^ |
| Childhood SES | .102 (.025)^***^ |  | .043 (.032) | .134 (.025)^***^ | −.003 (.036) | .091 (.032)^**^ |
| Own SES | .151 (.024)^***^ |  | .146 (.031)^***^ | .163 (.024)^***^ | .046 (.035) | .073 (.031)^*^ |
| SIMD | .146 (.024)^***^ |  | .131 (.031)^***^ | .044 (.078) | .054 (.035) | .159 (.031)^***^ |
| FEV | .174 (.029)^***^ |  | .181 (.036)^***^ | .106 (.029)^***^ | .042 (.041) | .246 (.036)^***^ |
| 6m Walk time | .110 (.024)^***^ |  | .107 (.030)^***^ | .073 (.024)^**^ | .021 (.034) | .164 (.030)^***^ |
| Grip strength | .195 (.038)^***^ |  | .245 (.047)^***^ | .129 (.037)^**^ | .071 (.054) | .202 (.048)^***^ |
| *APOE*† | −.097 (.053) |  | −.163 (.065)^*^ | .005 (.051) | −.064 (.073) | −.166 (.066)^*^ |
| BMI | −.020 (.024) |  | .038 (.030) | −.089 (.023)^***^ | .051 (.033) | .012 (.030) |
| Smoking† | −.020 (.047) |  | −.121 (.059)^*^ | .048 (.046) | .093 (.065) | −.140 (.059)^*^ |
| Alcohol | .081 (.029)^**^ |  | .077 (.035)^*^ | .068 (.028) | .060 (.040) | .048 (.036) |
| CVD† | −.018 (.055) |  | −.091 (.069) | .057 (.054) | .128 (.076) | −.224 (.069)^**^ |
| Hypertension† | −.123 (.047)^*^ |  | −.101 (.060) | −.102 (.046)^*^ | −.057 (.067) | −.130 (.060)^*^ |
| Diabetes† | −.226 (.083)^**^ |  | −.231 (.105)^*^ | −.229 (.082)^**^ | .045 (.117) | −.257 (.105)^*^ |

Note: * = *p* < .05; ** = *p* <.01; *** = *p* < .001; *p*-values uncorrected. † = categorical predictor; all other predictors continuous. Continuous predictors standardized with respect to both the predictor and the outcome; categorical predictors standardized with respect to the outcome only (analogous to Cohen’s *d*). *g* = general factor; SES = Socioeconomic Status; SIMD = Scottish Index of Multiple Deprivation; FEV = Forced Expiratory Volume in 1 second; *APOE* = *APOE* e4 carrier; Alcohol = alcohol g consumed per week; CVD = Cardiovascular Disease.

*Table S6*. Percentage change in predictor effect size after control for age 11 cognitive ability (comparing Tables S4 and S5) on the general factor of cognitive level and—in a separate model—the level each of the four cognitive domains. Data are shown only for results that were significant in Table S5.

| Covariate | General factor % change | Domain factor % change | | | |
| --- | --- | --- | --- | --- | --- |
|  | *g* level | Visuospatial  level | Crystallized  level | Verbal memory  level | Speed  level |
| Age (baseline) | −69% | −8% | −17% | −6% | −7% |
| Sex (female) † | - | −17% | −59% | −30% | - |
| Time lag | - | - | - | - | - |
| Age 11 IQ | - | - | - | - | - |
| Education | −52% | −57% | −47% | −62% | −69% |
| Childhood SES | −61% | −74% | −54% | −102% | −56% |
| Own SES | −58% | −53% | −55% | −80% | −67% |
| SIMD | −61% | −56% | −86% | −79% | −49% |
| FEV | −46% | −36% | −55% | −72% | −28% |
| 6m Walk time | −60% | −53% | −64% | −87% | −41% |
| Grip strength | −50% | −37% | −55% | −65% | −43% |
| *APOE*† | - | −4% | - | - | −1% |
| BMI | - | - | −52% | - | - |
| Smoking† | - | −46% | - | - | −83% |
| Alcohol | −67% | −62% | - | - | - |
| CVD† | - | - | - | - | −24% |
| Hypertension† | −30% | −33% | −24% | - | −26% |
| Diabetes† | −54% | −47% | −50% | - | −42% |

Note: † = categorical predictor; all other predictors continuous. *g* = general factor; SES = Socioeconomic Status; SIMD = Scottish Index of Multiple Deprivation; FEV = Forced Expiratory Volume in 1 second; *APOE* = *APOE* e4 carrier; Alcohol = alcohol g consumed per week; CVD = Cardiovascular Disease.

*Table S7*. Standardized associations of each predictor, all entered simultaneously, on the level and slope of decline in the general factors of level and slope and—in a separate model—in each of the four cognitive domains.

| Covariate | General factor estimate (SE) | |  | Domain factor estimate (SE) | | | | | | | |
| --- | --- | --- | --- | --- | --- | --- | --- | --- | --- | --- | --- |
|  | *g* level | *g* slope |  | Visuospatial  level | Crystallized  level | Verbal memory  level | Speed  level | Visuospatial  slope | Crystallized  slope | Verbal memory  slope | Speed  slope |
| Age (baseline) | **−.149 (.043)^**^** | .108 (.081) |  | −.130 (.057)^a^ | −.095 (.042)^a^ | −.140 (.067)^a^ | **−.179 (.058)^*^** | **.394 (.114)^*^** | .029 (.119) | .172 (.081)^a^ | .001 (.086) |
| Sex (female) † | .243 (.108) | **.578 (.192)^*^** |  | −.100 (.141) | .217 (.106)^a^ | .169 (.170) | **.409 (.144)^*^** | .181 (.271) | **1.162 (.295)^**^** | .427 (.203)^a^ | .384 (.211) |
| Time lag | −.002 (.042) | −.039 (.075) |  | .019 (.055) | −.016 (.041) | .037 (.065) | −.029 (.056) | .030 (.105) | −.125 (.112) | .016 (.079) | −.052 (.083) |
| Age 11 IQ | **.674 (.031)^***^** | −089 (.061) |  | **.461 (.042)^**^** | **.549 (.031)^**^** | **.602 (.052)^**^** | **.432 (.044)^**^** | −.187 (.088)^a^ | .038 (.091) | −.066 (.065) | −.074 (.067) |
| Education | **.224 (.036)^**^** | −.087 (.064) |  | **.135 (.047)^*^** | **.250 (.035)^**^** | **.178 (.055)^*^** | −.016 (.048) | −.095 (.092) | −.228 (.096)^a^ | .015 (.067) | .006 (.071) |
| Childhood SES | .015 (.032) | −.107 (.057) |  | −.013 (.041) | .039 (.031) | −.044 (.050) | .022 (.043) | −.032 (.079) | −.117 (.085) | −.145 (.060)^a^ | −.055 (.062) |
| Own SES | .082 (.035)^a^ | .074 (.061) |  | .113 (.045)^a^ | .062 (.034) | .013 (.053) | .060 (.046) | .134 (.088) | .029 (.092) | .054 (.065) | .039 (.068) |
| SIMD | .029 (.034) | .067 (.061) |  | .013 (.045) | .014 (.034) | −.012 (.053) | .099 (.046)^a^ | .031 (.087) | .045 (.092) | .030 (.065) | .051 (.068) |
| FEV | .095 (.043)^a^ | .097 (.077) |  | .067 (.057) | .070 (.042) | .026 (.067) | .144 (.058)^a^ | .230 (.109)^a^ | .018 (.114) | −.026 (.081) | .072 (.085) |
| 6m Walk time | .031 (.032) | .090 (.059) |  | .027 (.043) | .005 (.033) | .043 (.051) | .079 (.045) | .198 (.084)^a^ | −.023 (.088) | .010 (.063) | .068 (.066) |
| Grip strength | .062 (.054) | **.262 (.097)**^*^ |  | .147 (.071)^a^ | .055 (.053) | −.099 (.086) | .071 (.073) | −.049 (.135) | **.492 (.150)^*^** | .251 (.102)^a^ | .209 (.106) |
| *APOE*† | −.064 (.067) | **−.499 (.114)^**^** |  | −.212 (.085)^a^ | .082 (.064) | −.068 (.101)^a^ | **−.272 (.088)^*^** | −.349 (.163)^a^ | .091 (.173) | **−.357 (.122)^*^** | **−.440 (.127)^*^** |
| BMI | .021 (.033) | .053 (.058) |  | **.132 (.042)^*^** | −.077 (.031)^a^ | .097 (.049) | .089 (.043)^a^ | .057 (.079) | .013 (.084) | −.099 (.059) | .048 (.063) |
| Smoking† | .105 (.063) | −.180 (.111) |  | −.050 (.082) | .150 (.061) ^a^ | .136 (.097) | −.022 (.084) | −.224 (.157) | −.153 (.168) | −.076 (.119) | −.116 (.123) |
| Alcohol | .010 (.033) | −.069 (.059) |  | .006 (.043) | .007 (.032) | .025 (.052) | −.001 (.045) | −.219 (.083) | .027 (.088) | −.012 (.062) | −.048 (.066) |
| CVD† | .061 (.077) | −.073 (.138) |  | −.038 (.102) | .071 (.076) | .167 (.120) | −.054 (.104) | .078 (.195) | −.289 (.206) | −.070 (.147) | −.007 (.153) |
| Hypertension† | −.070 (.067) | .015 (.120) |  | −.047 (.088) | −.019 (.066) | −.131 (.105) | −.108 (.090) | −.132 (.168) | .196 (.177) | .151 (.126) | −.020 (.126) |
| Diabetes† | −.220 (.121) | −.440 (.213)^a^ |  | −.357 (.157)^a^ | −.033 (.118) | −.140 (.188)^a^ | −.340 (.162) | −.129 (.300) | −.628 (.321) | −.362 (.230) | −.260 (.236) |

Note: Bolded values are statistically significant. * = *p* < .05; ** = *p* <.01; *** = *p* < .001; all *p*-values corrected for False Discovery Rate. ^a^ = value was statistically significant at *p* < .05 before FDR correction. † = categorical predictor; all other predictors continuous. Continuous predictors standardized with respect to both the predictor and the outcome; categorical predictors standardized with respect to the outcome only (analogous to Cohen’s *d*). *g* = general factor; SES = Socioeconomic Status; SIMD = Scottish Index of Multiple Deprivation; FEV = Forced Expiratory Volume in 1 second; *APOE* = *APOE* e4 carrier; Alcohol = alcohol g consumed per week; CVD = Cardiovascular Disease.

*Figure S1.* ROC curves showing the prediction of attendance at wave 2 (left) and at wave 3 (right) from all of the potential predictors of cognitive decline (excluding the ‘lag’ variable) listed in Table S7. AUC = Area under the curve.
